# Frailty Screening in World Trade Center Responders: Validation of the WTC Clinical Frailty Index

**DOI:** 10.1093/geroni/igaf122.3938

**Published:** 2025-12-31

**Authors:** Chinmayi Venkatram, Ghalib Bello, William Hung, Olivia Alpizar, Hannah Thompson, Elena Colicino, Katherine Ornstein, Fred Ko

**Affiliations:** Icahn School of Medicine at Mount Sinai, New York, New York, United States; Icahn School of Medicine at Mount Sinai, New York, New York, United States; James J. Peters Department of Veterans Affairs Medical Center, New York, New York, United States; University of Massachusetts Amherst, Amherst, Massachusetts, United States; Icahn School of Medicine at Mount Sinai, New York, New York, United States; Icahn School of Medicine at Mount Sinai, New York, New York, United States; Johns Hopkins University, Baltimore, Maryland, United States; James J. Peters Department of Veterans Affairs Medical Center, New York, New York, United States

## Abstract

World Trade Center (WTC) responders are a unique cohort whose environmental exposures place them at increased risk for earlier onset of aging-related conditions, including frailty. Detecting frailty in this group is critical for guiding interventions to support healthy aging. The WTC Clinical Frailty Index (WTC FI-Clinical) is a continuous measure constructed from 30 health items routinely collected during responder monitoring visits. We aimed to use the frailty phenotype to validate and establish a meaningful cutoff for identifying frailty using the WTC FI-Clinical. We enrolled WTC responders aged 50 and older who received annual health monitoring at Mount Sinai’s WTC Health Program (WTCHP). Frailty was measured using both the frailty phenotype and WTC FI-Clinical. ROC (Receiver Operation Characteristic) analysis, with the frailty phenotype as reference, was used to assess discrimination and identify an optimal cutoff for the WTC FI-Clinical. Among 390 responders (median age 62, IQR 57–67), the frailty phenotype classified 54.1% robust, 40.8% pre-frail, and 5.1% frail. The WTC FI-Clinical demonstrated good discrimination for frailty (AUC 0.907, 95% CI: 0.858–0.952), and ROC analysis identified an optimal cutoff of 0.237, corresponding to 85% sensitivity and 76% specificity. Using this cutoff, 27.2% were classified as frail by the WTC FI-Clinical (versus 5.1% by the phenotype). These findings support its use as a practical screening tool to identify at-risk responders and guide healthy aging interventions within the WTCHP. It also extends frailty detection beyond traditional older cohorts, offering insight into how frailty can emerge earlier in populations with unique environmental exposures.

